# Standard Reference Material (SRM 1990) For Single Crystal Diffractometer Alignment

**DOI:** 10.6028/jres.106.058

**Published:** 2001-12-01

**Authors:** W. Wong-Ng, T. Siegrist, G. T. DeTitta, L. W. Finger, H. T. Evans, E. J. Gabe, G. D. Enright, J. T. Armstrong, M. Levenson, L. P. Cook, C. R. Hubbard

**Affiliations:** National Institute of Standards and Technology, Gaithersburg, MD 20899-0001; Inorganic 2, Lund University, Sweden; Bell Laboratories, Lucent Technologies, Murray Hill, NJ 07974; Hauptman-Woodward Medical Research Institute, Buffalo, NY 14203; Geophysical Laboratory, Washington, DC 20015; U.S. Geological Survey,, Reston, VA 22092; Steacie Institute for Molecular Sciences, NRC, Ottawa, Canada K1A 0R6; National Institute of Standards and Technology, Gaithersburg, MD 20899-0001; Oak Ridge National Laboratory, Oak Ridge, TN 37831

**Keywords:** alignment standard, Cr-content, international round robin, NIST SRM 1990, ruby spheres, single crystal x-ray diffractometers

## Abstract

An international project was successfully completed which involved two major undertakings: (1) a round-robin to demonstrate the viability of the selected standard and (2) the certification of the lattice parameters of the SRM 1990, a Standard Reference Material^®^ for single crystal diffractometer alignment. This SRM is a set of ≈3500 units of Cr-doped Al_2_O_3_, or ruby spheres [(0.420.011 mole fraction % Cr (expanded uncertainty)]. The round-robin consisted of determination of lattice parameters of a pair of crystals: the ruby sphere as a standard, and a zeolite reference to serve as an unknown. Fifty pairs of crystals were dispatched from Hauptman-Woodward Medical Research Institute to volunteers in x-ray laboratories world-wide. A total of 45 sets of data was received from 32 laboratories. The mean unit cell parameters of the ruby spheres was found to be *a*=4.7608 Å±0.0062 Å, and *c*=12.9979 Å±0.020 Å (95 % intervals of the laboratory means). The source of errors of outlier data was identified. The SRM project involved the certification of lattice parameters using four well-aligned single crystal diffractometers at (Bell Laboratories) Lucent Technologies and at NRC of Canada (39 ruby spheres), the quantification of the Cr content using a combined microprobe and SEM/EDS technique, and the evaluation of the mosaicity of the ruby spheres using a double-crystal spectrometry method. A confirmation of the lattice parameters was also conducted using a Guinier-Hägg camera. Systematic corrections of thermal expansion and refraction corrections were applied. These rubies_–_ are rhombohedral, with space group $R\bar{3}c$. The certified mean unit cell parameters are *a*=4.76080±0.00029 Å, and *c*=12.99568 Å±0.00087 Å (expanded uncertainty). These certified lattice parameters fall well within the results of those obtained from the international round-robin study. The Guinier-Hägg transmission measurements on five samples of powdered rubies (*a*=4.7610 Å±0.0013 Å, and *c* = 12.9954 Å±0.0034 Å) agreed well with the values obtained from the single crystal spheres.

## 1. Introduction

In order to provide industrial, academic and government laboratories with a Standard Reference Material^®^ (SRM) for the alignment of single crystal x-ray diffractometers, an international project was completed which involved two major undertakings: (1) an international round-robin to demonstrate the viability of the selected standard and (2) the certification of the lattice parameters of the SRM.

A lattice parameter standard is essential for the single crystal x-ray diffraction community for two principal reasons. First, x-ray structural determinations using automatic x-ray diffractometer data collection and automatic structure solution schemes require accurate initial cell parameter data. The unit cell metric gives a good indication of the Bravais Lattice, and therefore should be known as precisely as possible. Accurate cell parameters can only be obtained with well-aligned x-ray diffractometers; a standard crystal is critical for diffractometer calibration. Second, a standard is important for both intra- and inter-laboratory comparison of data. Much structural work reported in literature today is based on single crystal x-ray diffraction methods, and there have been claims of six digit accuracy in lattice parameters. The editors of Acta Crystallographica considered these data unrealistic and mostly unsupported. The Commission on Crystallographic Apparatus and Standards Committee of the International Union of Crystallography (IUCr) was requested to investigate the accuracy and precision of lattice parameters measured in the industrial, academic and government x-ray laboratories. The project was initially organized by Professor Ludmilla Malakhova of the Institute of Crystallography in the Soviet Union and passed into the hands of George DeTitta of the Hauptman-Woodward Medical Research Institute. An international round- robin project was conceived during the 1981 IUCr meeting in Ottawa, Canada. As various other institutions became interested in the project the goals of the project expanded and matured so that a number of important issues were identified. The American Crystallographic Association (ACA) also joined in this effort.

Presently, other than a few commercially available crystals from various manufacturers and materials that have been prepared locally at individual laboratories, no certified standard material is available for widespread use in diffractometer alignment. The leadership of NIST in this project is crucial to the development and the future distribution of the SRM for a number of reasons. First, it is the mission of NIST to take an active role in developing measurement standards and techniques, and NIST has a strong internal interest in producing SRMs for in-house research instruments as well. Second, NIST has the experience and expertise, in addition to the storage and distribution facilities required to make SRMs available for a wide range of users. It also has the necessary personnel to handle the business aspects of marketing and selling the products. Third, neither the IUCr nor the ACA has the resources to support a long term commitment to produce and maintain an SRM to supply a broad community of commercial, academic, and government needs.

This paper summarizes the round-robin and the SRM certification projects. The following discussion of the round-robin project includes the goals, procedures, results of statistical analysis, and errors in diffractometer alignment. Although the particular diffractometers used do not include all the types of diffractometers in use worldwide, this study gives a reasonable set of data for inter-laboratory comparison. The discussion of the SRM certification project includes descriptions of several important aspects, such as physical characteristics of the spheres, experimental procedures used for determining the lattice parameters, the Cr content of the ruby, and factors affecting accuracy of single-crystal diffractometer alignment and lattice parameter determination.

## 2. International Round-Robin

### 2.1 Goal of Study

The goal of this international round-robin project has four parts: (1) to determine realistic limits on the precision and accuracy of lattice parameters using various commercial diffractometers; (2) to assess the x-ray technique and the state of the instrument employed at each local laboratory, (3) to evaluate data collection method at each laboratory, and (4) to evaluate the usefulness of the ruby spheres as a NIST standard reference material (SRM) for diffractometer alignment.

### 2.2 Procedures

**Fig. 1:**
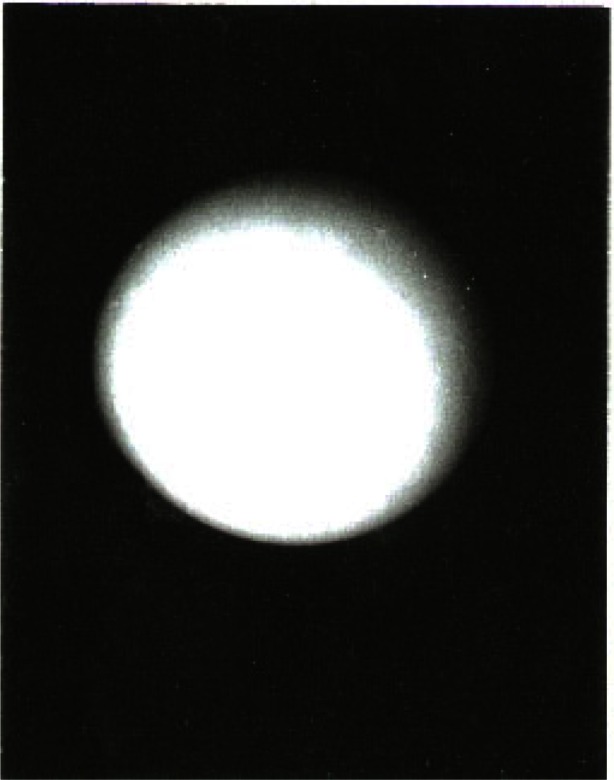
SEM micrograph showing the morphology of a typical ruby sphere (SRM 1991), with a diameter of 150 μm.

The round-robin project involves the use of single crystal x-ray diffractometers to determine the lattice parameters of a standard crystal (alignment standard) and an “unknown” reference crystal (representing a typical laboratory sample). Preliminary studies of the structural and physical properties of a batch of five hundred ruby spheres which were purchased by the ACA and IUCr from the Arcanum Corporation,^1^ Michigan, indicated these spheres to be stable, relatively homogeneous, easy to handle, and safe to use. A single crystal boule was prepared using the Verneuil technique (flame fusion) [1]. Cubes were cut from this boule by a diamond saw and then ground between two disks rotating in opposite directions to produce small spheres. These spheres were reported by the manufacturer to have a diameter of 0.152 mm and sphericity of 0.0013 mm. The sphere contains of the order of 3.4 × 10^14^ Al_2_O_3_ formula units. The high crystal quality and the high hardness give rise to small thermal motion parameters, and therefore produce strong reflections at high angles for MoKα radiation as well as for the copper. In addition, this radius of ruby sphere is appreciably smaller than a typical incident beam of a diffractometer and therefore can satisfy beam uniformity conditions. Figure 1 shows a SEM micrograph of a typical ruby sphere. These small crystals are nearly perfect spheres which allow accurate optical centering. From well-centered reflections at high angles, high precision lattice parameters can be obtained [2–5]. Therefore, they can be used as a round-robin standard and a potential Standard Reference Material^®^ for alignment of single crystal x-ray diffractometers.

**Fig. 2:**
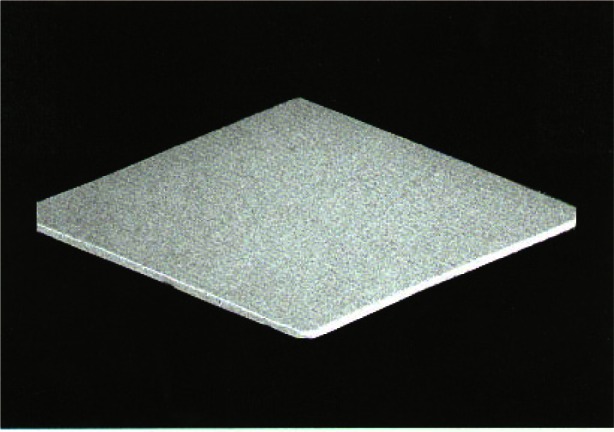
SEM micrograph showing the morphology of the zeolite crystal.

During the early stage of study, an organic crystal (Raffinose) was chosen as an unknown reference crystal which has cell dimensions ranging from moderately short to long (≈8×12×23 Å). This material, however, was found to be unstable for shipping and has been replaced by synthetic ferrierite zeolite crystals, which are stable under operating conditions. These “giant” zeolite molecular sieve crystals crystallized with a primitive orthorhombic unit cell, with a formula Al_2_Si_34_O_72_ · 2(C_5_H_5_N) · 2HF. These crystals exhibit a plate-like morphology, posing a typical challenge to crystal alignment. The cell parameters are estimated to be *a* = 18.8430(51) Å, *b* = 14.0981(33) Å and *c* = 7.4383(24) Å[6]. Figure 1 shows a SEM micrograph of a typical ruby sphere. These small crystals are nearly perfect spheres which allow accurate optical centering. Figure 2 shows a SEM micrograph of the morphology of a selected zeolite crystal. The zeolite crystals were obtained from the Chemistry Department of the University of Toronto, and were grown using non-aqueous solvent [6].

**Fig. 3:**
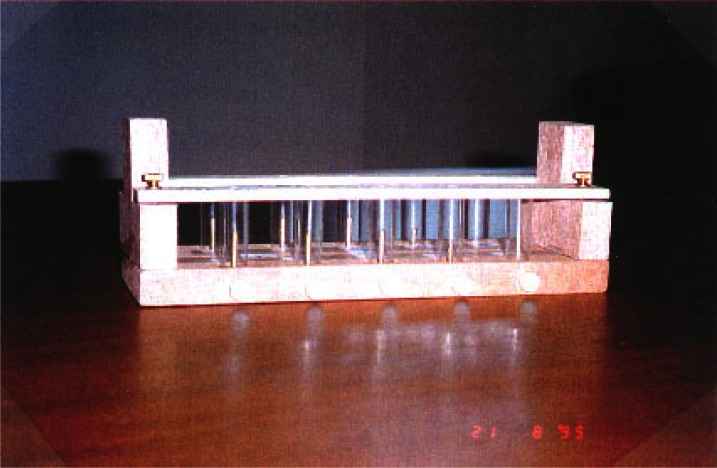
Interior of the crystal kit.

Optical examination of crystals of both ruby and zeolite was followed by mounting approximately 100 of each type. Both the zeolite and ruby spheres were mounted on the tips of fibers of approximate diameter of 0.1 mm which were secured in blocks. The zeolite crystals were mounted along the face diagonals. Preliminary characterization, both by x-ray photography and by diffractometry methods (using Siemens P2_1_ diffractometers) allowed identification of the most suitable candidates for the round-robin project. Orientations were measured for the selected round-robin crystals. Reflections were measured on the positive and negative sides of 2*θ* and in eight positions described by Hamilton in the International Table for Crystallography [2], by King and Finger [3] and by Hazen and Finger [4]. Typically 48 reflections were used (6 independent reflections in 8 quadrants). A total of 100 crystals (50 sets of ruby spheres and zeolite crystals) were used to prepare the round-robin kits. Ten kits each containing 5 rubies and 5 unknowns (Fig. 3) were assembled and shipped to participants from the Hauptman-Woodward Medical Research Institute, Buffalo. Mounts of the crystals were carefully evaluated as to mechanical strength. Special boxes were constructed for the shipment of the crystals. Several severe baggage-handling simulations were undertaken (with drops of 2 to 3 meters) to evaluate the likelihood that the samples would survive the shipping process. The samples were assembled in their enclosures package and mailed out. Notebooks were prepared with necessary data on the crystals, literature references, instruction sheets, etc. and were dispatched along with the samples for the evaluation of diffractometry. Instructions were designed to ensure the collection of data pertinent to the evaluation of the crystal centering algorithm and the alignment of the instrument. This procedure was based on the method developed by King and Finger [3].

The main working hypotheses of the project is that the main errors associated with obtaining accurate lattice parameters are due to the misalignment of the diffractometer and of the diffracting samples. Therefore participants were expected to measure data pertinent to evaluation of: the crystal centering algorithm, the alignment of the instrument, and the lattice parameter determination software. The procedure included in the round-robin instructions also called for measurement of auxiliary data in order to determine the mechanical and optical conditions of sample and diffractometer.

In summary, scientists were asked to perform the following:

1. Determine the lattice parameters of the zeolite crystals by standard laboratory procedures in use in their facilities.
2. Determine additional diffraction data (i.e., orientation matrix [7]) as instructed, providing information concerning the state of their equipment and sample.
3. Measure data on the ruby standard pertinent to the evaluation of the centering algorithm, alignment of the instrument, and lattice parameter determination software.
4. Submit results for statistical analysis.

After receiving the data, the design team which included scientists from the Geophysical Laboratory, the Hauptman-Woodard Medical Research Institute and NIST, then analyzed results statistically.

**Fig. 4:**
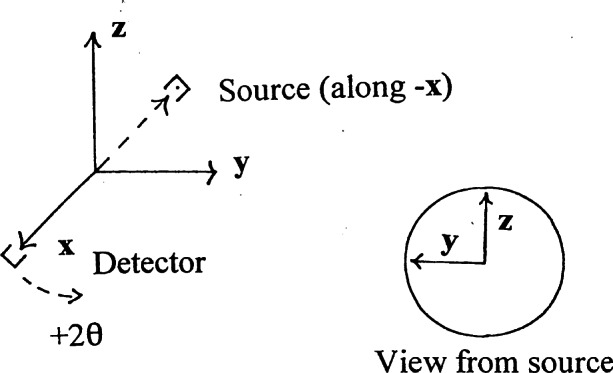
Orthonormal coordinate frame used for analysis.

The orientation matrices used for the evaluation were determined with respect to the following orthonormal coordinate frame (shown in Fig. 4), where *x, y*, and *z* are the crystal Cartesian axes, and a*, b*, and c* is the reciprocal lattice. This orthonormal coordinate frame is valid for diffractometer types such as Syntex, Nicolet, Siemens *P*3, etc.

$$UB=\left(\begin{array}{ccc} a*x & b*x & c*x\\ a*y & b*y & c*y\\ a*z & b*z & c*z \end{array}\right)$$

where ***UB*** is the orientation matrix

$$\left(\begin{array}{c} ho\\ ko\\ lo \end{array}\right)=UB\left(\begin{array}{c} h\\ k\\ l \end{array}\right)$$

where *h, k, l* are the Miller indices, and *ho, ko, lo* are the coordinates of a reflection in the *ϕ* axial frame.

The definition of diffractometer angles used is that given by Busing and Levy [5], which is the bisecting mode in which the incident, diffracted beams and the counter all lie on a horizontal plane, and *ω* = 0°.

Various types of systematic errors can affect the positions of the diffracted beam, which in turn affect the lattice parameters of the crystal being studied. These errors include diffractometer zero position, errors in crystal centering, and misalignment of the instrument (including error in counter or tube height). In the procedure by King and Finger [3], the measured angles for a single reflection, or Friedel pair of reflections in eight different orientations (quadrants) are used to determine the values for various errors associated with the mounting of the crystal and alignment of the diffractometer.

### 2.3 Participants

A survey of structural crystallographers identified about 50 laboratories worldwide who were interested and willing to participate in the round-robin project, and preliminary information provided by this survey has also identified the kinds of equipment that were in use in the community for this work.

The 50 international participants are all active crystallographers who make frequent use of diffractometers for their research, the areas of macromolecules, small molecules, inorganic, organic, intermetallic, pharmaceutical, and ceramics. More than 10 different types of diffractometers were employed by these participants, including CAD4 (Enraf Nonius), AFCS6 (Rigaku), P3 (Syntex), SMART (Siemens CCD), R3m (Nicolet), P21 (Syntex), Huber, Kumar, StoeAED, Stoe-4C, R-4Cir, and P4. Molybdenum radiation (MoKα) was used by most of the laboratories, followed in frequency of use by copper radiation (CuKα). Silver radiation (Ag Kα was used in one laboratory.

### 2.4 Results and Discussion

#### 2.4.1 Lattice Parameter Measurement

A total of 45 (44 complete) sets of reports for the ruby spheres and zeolite crystals have been received from 32 laboratories. Information obtained from these reports includes type of diffractometer(s) and wavelength used, identification of crystal sets, measured cell parameters, and relevant angles of the diffractometers including in the Eulerian system: *χ, ϕ, ω*, and 2*θ* [3]; and in the case of a Kappa-type diffractometer, the angles are expressed in the Kappa system. Among these data sets, 15 laboratories used the eight-quadrant method, which allowed for further detailed evaluation by using the routine by King and Finger [3].

Most of the participants collected their data within the ambient temperature range. A correction of the lattice parameters to nominal room temperature of 298 K was made before comparison. Belyaev [8] and Campbell and Grain [9] reported the thermal expansion coefficient of Al_2_O_3_. Within the range of 0 °C to 100 °C the α-Al_2_O_3_ was found to expand approximately linearly. The expansion is anisotropic and the corrections can be calculated according to the following expression:

$$\begin{array}{l} a^{\prime }(25{}^{\circ}C)=a[1+\alpha_{a}(25-T)],\;\text{where}\;\alpha_{a}=5.0\times 10^{-6}\\ c^{\prime }(25{}^{\circ}C)=c[1+\alpha_{c}(25-T)],\text{where}\;\alpha_{c}=6.66\times 10^{-6}. \end{array}$$

One participant collected the data at a low temperature of 153 K, and the data were not used.

We have also applied a refraction correction [10] in two parts. One corresponds to the Snell’s law correction and is too small to be included (δ*d/d* = −*v* cot*θ* = (1−*n*) cot*θ*). Another part is due to the change of wavelength: 1−*n* = *cρλ*^2^ (Σ*Z*/Σa), where *ρ* is the density (taken as 3.98 g/cm^3^ [8]), *λ* is the wavelength, *Z* is the atomic number, *a* is the atomic weight, and *c* is a constant. In the equation, δ*d/d* = (1−*n*)/*n*, where *n* is the refractive index, we found that for Mo radiation (1−*n*) = 2.69 × 10^−6^, and for Cu radiation, the value of (1−*n*) = 1.27 × 10^−5^. This correction is a relatively small quantity.

For the ruby crystals, most least-squares lattice refinements were constrained to obey the hexagonal setting. For those that were not constrained, results indicated that the parameters are very close to hexagonal. Table 1 summarizes the lattice parameters of the ruby spheres after the application of thermal expansion and refraction corrections. For those laboratories that have reported more than one set of data, the values were averaged and the mean value obtained was then computed with other data sets for obtaining the global mean. Table 1 also gives the mean lattice parameter values for several laboratories. Table 2 gives the corresponding data for the zeolite crystals. The mean values of the lattice parameters and the 95 % intervals on the grand mean and on the population of laboratory means are:

1. ruby spheres:
  $$\begin{array}{l} a=4.7608\;Å\;\pm 0.0011\;Å(95\%\;\text{intervals on grand mean})\\ \;\pm 0.0062\;Å(95\%\;\text{intervals of laboratory means})\\ b=4.7609Å\;\pm 0.0010Å\\ \;\pm 0.0057Å\\ c=12.9979Å\pm 0.0035Å\\ \;\pm 0.020\;Å \end{array}$$
2. zeolite crystals:
  $$\begin{array}{l} a=18.8338\;Å\;\pm 0.0051\;Å(95\%\;\text{intervals on grand mean})\\ \;\pm 0.014\;Å\;(95\%\;\text{intervals of laboratory means})\\ b=14.1036\;Å\;\pm \;0.0054\;Å\\ \;\pm 0.014\;Å\\ c=7.4366\;Å\;\pm 0.0026\;Å\\ \;\pm \;0.0070\;Å \end{array}$$

The mean value of the lattice parameters in general agrees well with the certified SRM data (discussed later), which is (at 25°C): *a*=4.76080Å±0.00029Å (expanded uncertainty), and *c*=12.99568Å±0.00087Å, despite the large spread in reported values.

The fact that the round-robin results show a large spread (as compared to the certified values) of data indicates that uncertainty of measurements in general laboratories is relatively large unless great care is taken about the diffractometer alignment. The closest match of the lattice parameter results between the round-robin data and ruby SRM value is that submitted from laboratory No. 9 (*a* = 4.7609 Å, *c* = 12.9951 Å).

#### 2.4.2 Histograms

**Fig. 5:**
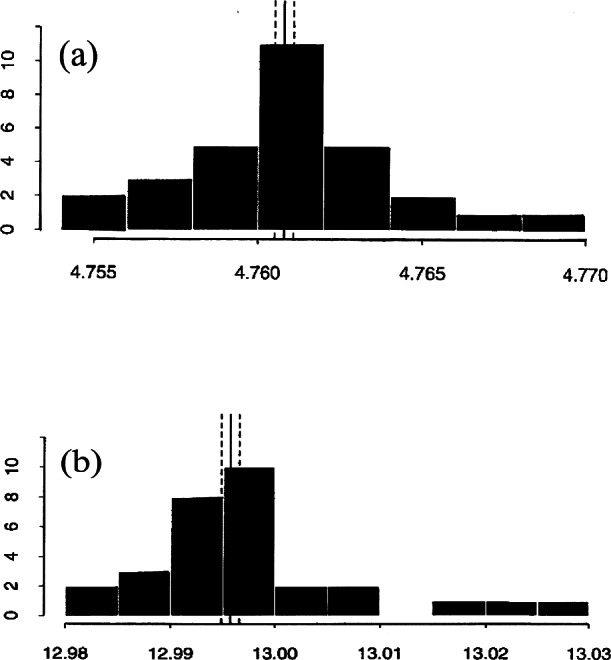
Histograms of the results of (a) lattice parameters *a*, and (b) lattice parameter *c* of the ruby spheres from 30 laboratories participating in the round-robin project.

Figure 5 displays histograms of the results of lattice parameters *a* and *c* of 30 laboratories participating in the round-robin project. Note that results of two laboratories were not used due to problems with the measurements. For the lattice parameter *a*, the results of the laboratories are centered on the certified value and are symmetric around this certified value. For the lattice parameter *c*, there are three results that are somewhat separated from the other results. The spread of the results for the *c* parameter (relative standard deviation = 0.0035) is greater than that for the *a* parameter (relative standard deviation = 0.001).

#### 2.4.3 Youden Plots

Those laboratories that reported ruby lattice parameters with relatively large deviations from the mean also reported corresponding significant deviations for the zeolite crystals (for example, laboratory Nos. 14, 24, and 28). This similarity strongly indicates that the large deviation of the ruby lattice parameters can be used as an indicator of the alignment condition of the diffractometer. Once the diffractometer is re-aligned using the ruby spheres, the accuracy of the lattice parameters of future determinations will hopefully be improved.

**Fig. 6:**
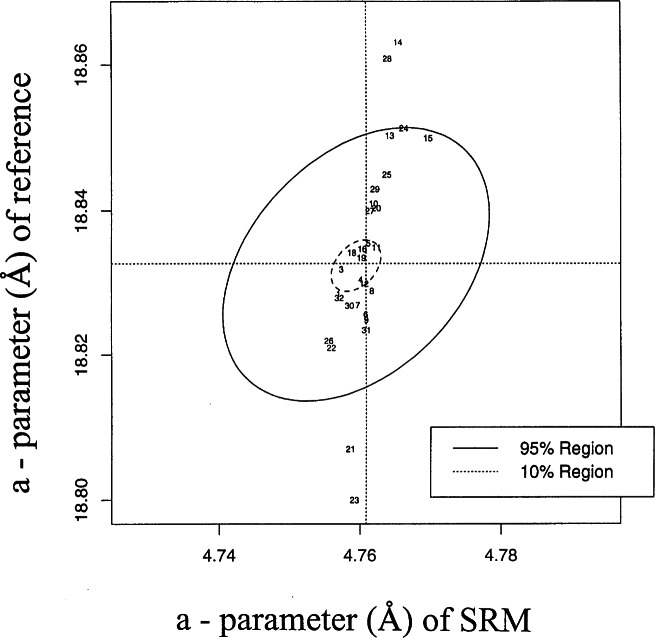
Joint confidence regions on the round-robin results (Youden analysis). Plot of lattice parameter *a* from the reference material (zeolite crystals) against the lattice *a* of the standard reference material (ruby spheres).

One technique to analyze results from a round-robin exercise is the use of the Youden plot [11]. In the Youden plot, two related measurements are plotted versus each other. In the present study, the lattice parameter *a* from the reference material is plotted against the lattice a of the standard reference material (see Fig. 6). The plot and auxiliary calculations can be used to quantify between-laboratory variation (laboratory biases) and within-laboratory variation. A laboratory is biased if it tends to be higher or lower than the true value. The presence of laboratory biases appears in the Youden plot as a positive linear pattern.

Using robust measures of the mean and standard deviation of the results, outlier laboratories can be identified [12]. The two dotted lines in Fig. 6 represent the robust estimates of the means of the two sets of measurement results. The solid-lined ellipse defines a 95 % confidence region for the pairs of results. Four data sets (laboratories Nos. 14, 21, 23 and 28) are well outside the ellipse.

**Fig. 7:**
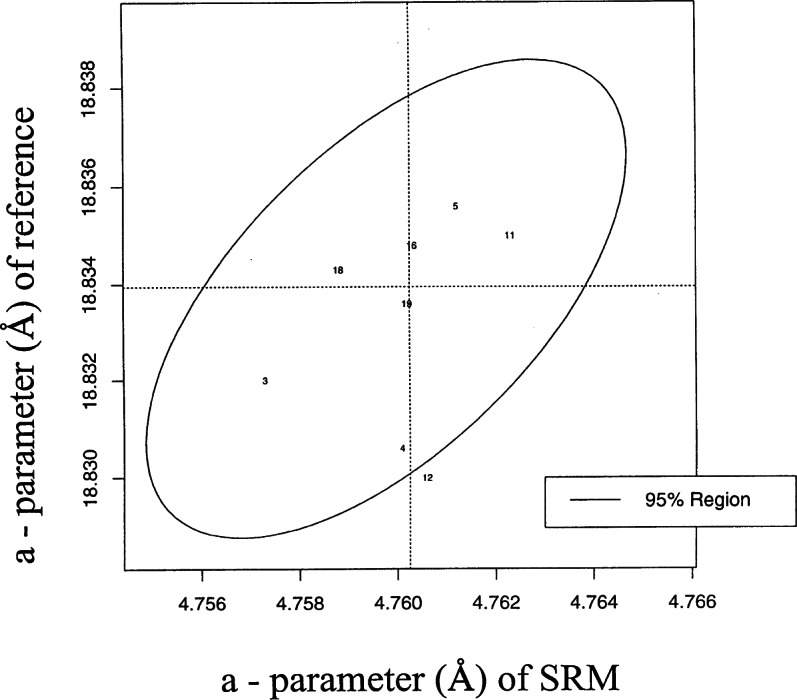
Youden analysis based solely on the results of eight best laboratories.

The dotted-line ellipse in Fig. 6 defines a 10 % confidence region. Using this region, the best data sets determined at eight laboratories are determined. Figure 7 repeats the Youden analysis based solely on these eight laboratories. One of the eight is just outside the 95|jpercnt| region, which is not indicative of outliner behavior. Even for these “best” data sets, between-laboratory variation exists. The sum of the between- and within-laboratory variation can be used as the basis of a measure of the standard uncertainty for these laboratories. The resulting relative standard uncertainty is 0.0018. Thus, the third decimal place is the limit of the accuracy for these “best” data sets.

#### 2.4.4 Errors of Diffractometer Alignment

As mentioned above, various types of errors could be evaluated for the data sets that were collected using the eight-quadrant technique, namely, diffractometer zero position, errors in crystal centering, and misalignment of the instrument including error in counter or tube height. In general, for data that fall well within the region of the 95 % interval, these errors appear to be relatively small. On the other hand, those data sets, which show great deviations from the mean also exhibit significantly large errors due to one or more types of misalignment error.

**Fig. 8:**
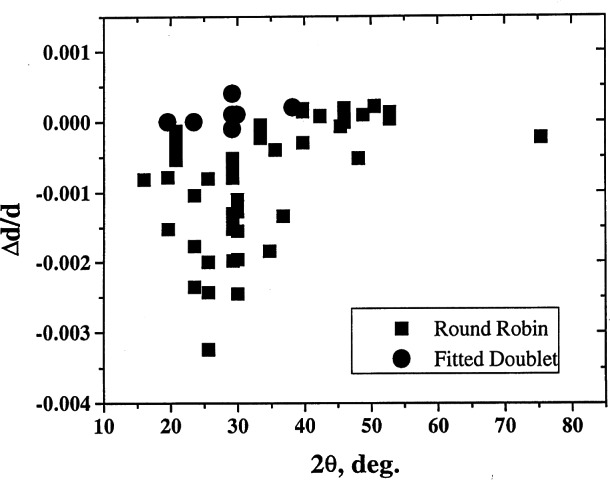
Plot of δ*d/d* vs 2*θ*.

**Fig. 9:**
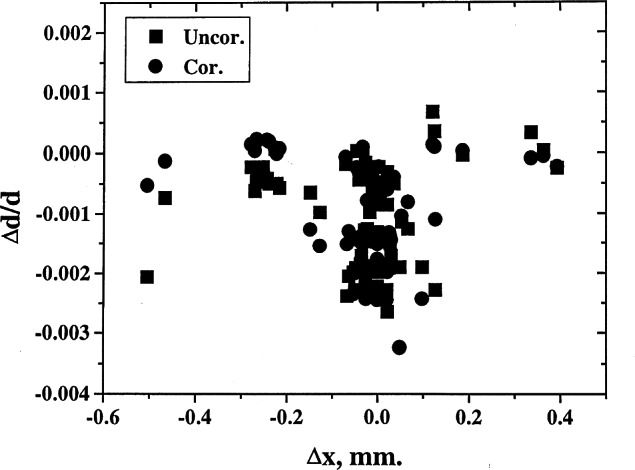
Plot of δ*d/d* vs Δ*x* crystal offset.

**Fig. 10:**
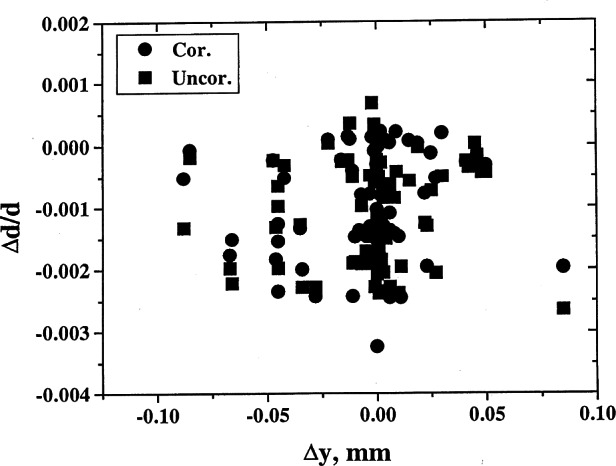
Plot of δ*d/d* vs Δ*y* crystal offset.

**Fig. 11:**
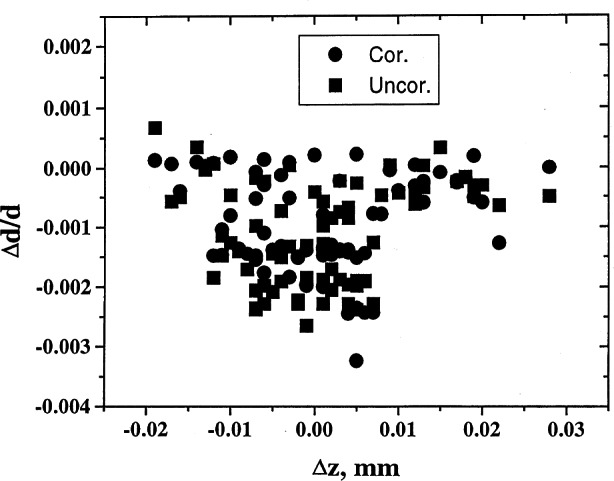
Plot of δ*d/d* vs Δ*z* crystal offset.

**Fig. 12:**
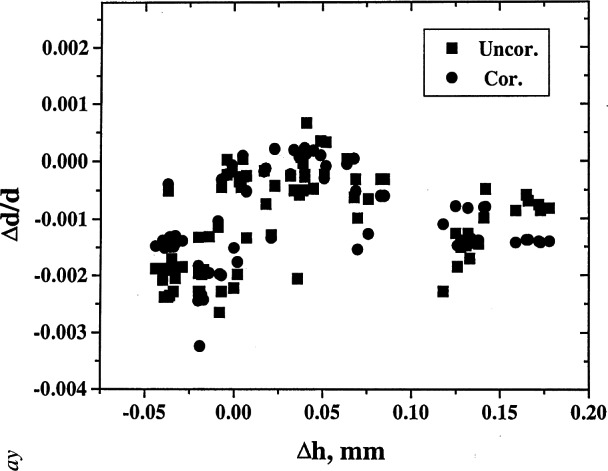
Plot of δ*d/d* vs Δ*h*, an error in the height of the counter aperture [3].

On many occasions, deviations of measured results from standard values could be explained in terms of the errors in the diffractometer alignment. For example, one can plot the values of δ*d/d* versus various types of errors. Estimation of the angles can be used to correct for the errors from the measurements of the ruby spheres. The corrected values can then be used to refine the lattice parameters, and much higher precision can be obtained. Examples of results of analysis pertaining to various types of corrections are shown in Figs. 8–12. Fig. 8 shows the Δ*d/d* values (where Δ*d* = *d*_std_−*d*_obs_) versus 2*θ*. The round-robin data are shown as filled squares. The α_1_−α _2_ doublet (with latest values of Mo and Cu wavelength reported by Haertwig et al. [13], i.e., *λ* for CuKα is 0.154059292(45) nm, *λ* for MoKα is 0.070931631(84) nm) was used for the fitting. It is seen that the fitted doublet values (filled circles) almost form a straight line at the value of Δ*d/d* = 0.0. It can be seen that most of the measured reflection data have relatively low 2*θ* values which are lower than the fitted doublet values.

Figures 9–12 contain the plots of Δ*d/d* versus Δ*x*, Δ*y*, Δ*z*, and Δ*h* which are the errors in centering the crystal on the diffractometer. For ruby spheres, these deviations should be no larger than ±0.005 mm; however, the value was found to be as large as 0.5 mm for Δ*x*. Clearly, such large apparent errors are indicative of severe problems with the diffractometer, including large inaccuracies and/or non-uniformity in the gears, or failure of the goniometer axes to intersect in a point, or lack of centering of the crystal.

There were a few examples of “bad” lattice parameter data sets that we have examined and we were able to identify the source of the “problem.” Three examples of measurement of the ruby spheres can be used to illustrate this point (laboratory Nos. 14, 15, and 28). Each of these laboratories used the eight-quadrant routine. For each set of data, the maximum deviation between dcal and corrected dobs is −0.002 Å. It appears that the corrections succeeded in removing the main errors. For No.28, the value of Δ(*h*) ranged from 0.125 to 0.142. This large value indicates a serious misalignment of the diffractometer, due to the counter aperture offset. The corresponding Δ(*h*) value of No. 14 was from 0.069 to 0.118, for No. 15 is from −0.009 to −0.037, which is approximately the resolution of the measurement.

### 2.5 Summary of the Round-Robin Study

The results of the round-robin project re-emphasized that well-aligned diffractometers are essential for obtaining accurate lattice parameters, and confirmed that the ruby spheres satisfy the criteria required of a standard reference material.

1. The ruby spheres are a stable material that possesses high symmetry. They are easy to handle and readily used to perform both optical and diffractometer alignment, and are a good standard for alignment, and for inter-laboratory comparison of data. They can be useful to diagnose various possible sources of diffractometer errors (including misalignment of the crystal in *x,y,z* coordinates, zero settings for the instrument, error in counter or tube height, etc.), therefore enabling accurate alignment of the instrument.
2. The magnitude of the deviation between the measured ruby lattice parameters and the SRM values can be used as an indicator for the condition of the diffractometer. Once the diffractometer is re-aligned using the ruby spheres, the accuracy of the lattice parameters from determinations of unknown crystals will be improved.
3. The mean value of the lattice parameters of the ruby data sets agree with the SRM data and that reported by Kuperman et al. [6], respectively, despite the large spread of round-robin data.
4. For data sets with large deviations of the measured ruby cell from that of the SRM value, the corresponding cell data of the zeolite crystals also show a similar magnitude of deviations from the known values, indicating that improved alignment using the ruby will increase the accuracy of a laboratory sample.
5. With the exception of a few outlier results, the distributions for both the ruby and zeolite data sets are symmetric. 6. To obtain accurate cell parameters, high angle reflections must be used (i.e., 2*θ* > 60° (Mo), peak separation of α_1_, α_2_ ≈ 0.4°) for least-squares refinements, otherwise the lattice parameters are too small.
6. The eight-quadrant algorithm is a reliable method to be used for single crystal diffractometer alignment, and the King and Finger method [3] can be used to estimate angle corrections.
7. In some data sets, correction of diffractometer or crystal alignment errors resulted in much better agreement with the SRM value. In other cases, no single variable can adequately account for the variations, and there may be inherent diffractometer defects.
8. The standard uncertainty from the results of eight “best” data sets is 0.0018. Thus, the third decimal place is the limit of the accuracy for these “best” laboratories. A realistic limit for uncertainty obtainable in laboratories is estimated to be Δ*a/a* > 2 × 10^−5^. Any value smaller than this reported in the literature should be regarded as questionable.

## 3. Certification of the Ruby Spheres (SRM 1990)

### 3.1 Technical Objective

The technical objective of this project is to provide industry, academic and government laboratories with a standard reference material (SRM) for the alignment of single crystal diffractometers. This SRM is intended to improve the accuracy of lattice parameter determinations, and can be used to evaluate the x-ray technique and the state of the instrument employed at each local laboratory. The auxiliary data on the chromium content will also be useful for microanalytical calibrations.

### 3.2 International Collaborations

The development of the SRM has been carried out as a team effort, involving NIST, Lucent Technologies, Woodward-Hoffmann Medical Research Institute, Geophysical Laboratory, National Research Council (NRC) of Canada (Ottawa), U.S. Geological Survey, and Oak Ridge National laboratory.

Bell Laboratories (Lucent Technologies) and NRC of Canada have state-of-the-art, well-aligned serial diffractometers equipped with advanced data collection and analysis software necessary for the completion and certification of SRM 1990. A Guinier-Hägg camera was available at the U.S. Geological Survey and was used to perform secondary measurements by crushing the spheres into powder. NIST has a well-aligned single crystal x-ray diffractometer for orientation determinations of the spheres, as well as electron microprobe and scanning electron spectroscopy/energy dispersive x-ray (SEM/EDS) equipment for the determination of the Cr-content of these spheres.

### 3.3 Characteristics of the Ruby Spheres

An additional 3000 ruby spheres were purchased from the Arcanum corporation for the SRM certification process. Crystals for the round-robin and the SRM projects were obtained from the same boule to ensure maximum homogeneity.

In order to confirm that the Cr^3+^ ion substitutes for an Al^3+^ ion in ruby and to understand the local structural arrangement of ions around a substituted Cr^3+^ ion, Kizler et al. studied extended x-ray absorption fine structure (EXAFS) in the vicinity of the Cr absorption edge [14]. The findings were compared by Mott-Littleton [15] with an ionic model using two sets of pairwise potentials. Both the EXAFS results and the computations reveal that when Cr^3+^ ion substitutes for an Al^3+^, which is smaller, the surrounding ions relax to an arrangement similar to that for Cr in α-Cr_2_O_3_. For example, the octahedra of oxygen ions surrounding the Cr^3+^ ion is expanded, becoming similar in size to that characteristic of α-Cr_2_O_3_.

These ruby spheres fulfill the requirements for a standard, namely, they are a readily available material that has long term chemical stability and no phase transformation over a wide range of temperature. Ruby is insoluble in most solvents and not subject to radiation damage. It is also non- toxic, adequately homogeneous (less than 0.02 % mole fraction variation) and with a very small mosaic spread of 0.005° to 0.015° full-width-half-maxima (FWHM), which will give rise to properly shaped reflection profiles (discussed later). These spheres also possess high symmetry (rhombohedral) with the space group of $R\bar{3}c$ (Al and Cr in position 12*c*: 0,0,*z*; and O in position 18*e*: *x*,0,1/4 [16]). High symmetry will allow comparison of many symmetry-equivalent reflections. In addition to many reflections of high intensity which can be used for relatively fast measurements, ruby has a relatively low absorption coefficient of 124 cm^−1^ (for Cu radiation), which enables valid comparison of intensities of symmetry equivalent reflections.

### 3.4 Experimental

The lattice parameters of SRM 1990 were studied and certified by using four well-aligned commercial single crystal diffractometers (three Enraf-Nonius CAD4 and one Picker). A second method, the Guinier-Hägg transmission technique, was employed to support the diffractometer single crystal lattice parameter data. Statistical analysis of the resulting data was carried out in collaborations with statistician Mark Levenson of the Statistical Division of NIST. Auxiliary data such as the content of the chromium in these crystals were analyzed using the electron microprobe, and also by the energy dispersive x-ray technique (EDS).

#### 3.4.1 Measurements using Single Crystal Diffractometers

The ruby crystals were mounted with a minimum amount of epoxy on the tip of ≈0.1 mm glass fibers or Lindemann capillary tubes. Among them, two sets of measurements (11 and 15 spheres each) were studied using Enraf-Nonius CAD4 diffractometers equipped with graphite monochromatized Mo and Cu radiation, respectively, at Bell Laboratories (Lucent Technologies). At NRC, fifteen spheres were studied using a CAD4 diffractometer equipped with monochromatized CuKα radiation, and four with a Picker Diffractometer equipped with monochromatized MoKα radiation. The diffractometer control program, DIFRAC [17], which has state-of-the-art data collection and data reduction schemes was employed for all data collection and reduction. This software package was developed at the National Research Council (NRC) of Ottawa and was described at the 1994 ACA Summer Meeting, Abstract M14 [18]. This program can be adapted to machines with different geometry, including the Kappa geometry used by the CAD4 diffractometers. All angles are specified in terms of the Eüler geometry with Eüler angles, 2*θ, ω, χ*, and *ϕ*.

On a CAD4 machine all precise centering of peaks is achieved by optimizing 2*θ, ω, χ*, at fixed *ϕ*, with continuous slow scans in the following sequences: (1) an *ω*/2*θ* scan with a variable slit, (2) a 2*θ* scan with −45° slit, and (3) a 2*θ* scan with the +45° slit. From the centroids of these scans the optimum values 2*θ, ω, χ*, and *ϕ* which will center the peak in the detector are calculated. If the initial position of the peak is vastly displaced from the center of the detector the routine performs an initial step scan in 2*θ, ω*, and *χ* to improve the starting position for the final precise adjustment. Peaks from an unknown crystal are located by rotating *ϕ* through 180° at each point on a specified grid of locations in 2*θ*, and *χ*, until the required number of coarse peak positions has been found and saved. These positions are then subjected to the precise centering described above and the final positions are used by the indexer to find the orientation matrix and the best reduced cell. The program also includes 2*θ*_0_, *ω*_0_, and *χ*_0_ corrections. The centroids of the peaks were found by determining the high and low-angle half-heights of fully resolved a_1_ peaks, followed by integrating between the two actual positions used to find the median. In this way, peak asymmetry will also be accounted for. Crucial to Kα_1_–α_2_ doublet is that the dispersion from the monochromator is at right angles to the scattering plane (vertical dispersion from monochromator and horizontal scattering plane for the CAD4 and the Picker diffractometers). Then, the Friedel pairs are symmetrical and the splitting of the doublet is identical, giving Friedel centroids relative to 2*θ*_0_ = 0 is determined unambiguously. The resolution of the doublet depends on the beam divergence and the size and mosaicity of the sample. The primary beam divergence is tied to the monochromator mosaic and the collimation of the beam. The ruby then samples 0.15 mm of the beam at the center. Therefore, the diffracted beam profile is a convolution of the monochromator mosaic (≈0.05° to 0.3°), the collimator, the ruby diameter and the ruby mosaic. The ruby mosaic is much smaller than the monochromator mosaic and therefore does not contribute. For each input reflection +*h*, +*k*, +*l* and −*h*, −*k*, −*l* are centered together with symmetry equivalents. The influence of absorption can be further reduced by measuring the difference in±for the Friedel reflections (Bond method), which is related to 2*θ*.

Because of the Kα_1_–α_2_ doublet, in order to obtain accurate *d*-spacing values, high 2*θ* angles were employed for the determination of lattice parameters in order to avoid α_1_ and α_2_ overlapping. For CuKα radiation, the 2*θ* values should be greater than 120° (at 120°, assuming FWHM of a peak profile of 0.3°, the α_1_–α_2_ splitting is 0.51°. This is a good separation and a valid determination of the α_1_ peak position by using the Busing and Levy method [19] (step to half-intensity on both sides of peak top)). For MoKα radiation, at 120° 2*θ*, α_1_/α_2_ separation is 1.2°; at 60°, of 0.4° separation (α_1_- of 59.936°, and α_2_ of 60.336°). Therefore, for Mo radiation, it would be important to use reflections with 2*θ*> 60° for obtaining accurate lattice parameters.

For precise instrument alignment, at Bell Laboratories, equivalent settings of selected reflections were obtained in all octants for data sets collected by using Mo radiation in order to establish zero corrections on 2*θ, ω*, and *χ*. Using the diffractometer equipped with Cu radiation, not all equivalent reflections were accessible at high angles, therefore 2*θ*_0_ corrections were applied by using the software UNITCELL [20] which determines the unit cell parameters by incorporating 2*θ*_0_ as a parameter. In the case where “error-free” 2*θ*-values were obtained, the final lattice parameter refinement used the 2*θ*’s only.

X-ray wavelengths used for all calculations were taken from Haertwig et al. [13] and from Cohen and Taylor [21]. The values for CuKα_1_ maximum is 1.54059292 (45)Å or (8047.8264(24) eV, and for MoKα_1_ radiation the maximum is 0.70931631 (84) Å, or 17479.401 (21) eV.

#### 3.4.2 Measurement of the Cr Content

The homogeneity and the quantity of the chromium content of these spheres were investigated by using both electron microprobe and quantitative SEM/EDS techniques. A total of 15 spheres were studied. One of the spheres was randomly selected to study in detail using the electron microprobe technique for the Cr concentration, and to study whether Cr is relatively uniformly distributed in the sphere. After the Cr content was obtained, this sphere was in turn used as a secondary standard for the rest of the 14 spheres by using an SEM/EDS broad-beam technique. These spheres were prepared for analysis by potting in epoxy and polishing and carbon coating. The final polishing step was completed using 0.1 μm diamond abrasive.

##### 3.4.2.1 Electron Microprobe Study of the Secondary Standard

###### Sample Preparation

Polished grains of chromite and Cr-bearing pyroxene mineral standards [22] were used for the evaluation of the Cr content. These two standards and the ruby sphere were mounted on a one- inch diameter holder. The sample block was carbon-coated for analysis using standard laboratory procedures. The sample block was mounted in the electron microprobe stage holder along with laboratory reference standard blocks.

###### Analytical Method Summary

The samples and laboratory reference standards were placed in a JEOL 8600 electron microprobe and examined by reflected light optical microscopy, secondary and back-scattered electron imaging, qualitative x-ray microanalysis using an energy dispersive x-ray detector, and quantitative x-ray microanalysis using a wavelength dispersive x-ray detector (WDS). Standard methods of examination and analysis were employed [23]. Analyses were performed at a electron beam accelerating potential of 15 keV and a current of ≈30 nA. The electron microprobe was calibrated to perform quantitative DS analysis using the Al Kα and Cr Kα x-ray lines. Peaks and background positions for the Al and Cr x-ray lines were determined by performing wavelength scans over the peak and background regions in laboratory standards of Cr_2_O_3_ ad Al_2_O_3_ (primary standard). A hypersthene sample from the Smithsonian was analyzed for Cr and used as a secondary standard. Three replicate analyses were performed on standard hypersthene and 16 replicate analyses were performed on the ruby pellet. Data was processed using the *ϕ*(*ρ* z) correction of Armstrong [23] with the correction program CITZAF [24].

##### 3.4.2.2 EDS Analysis of the Ruby Spheres

The secondary standard was used for analysis in the remaining 14 ruby spheres. For this, samples were carbon coated and analyzed in an AMRAY 1400 SEM, with the accelerating potential set at 15.8 kV, as confirmed by measurements of the upper energy limit of the continuum. Beam current, measured with a Faraday cup, was maintained at 1.0 nA. Sample inclination was 45° and x-ray take-off angle was 41°. The secondary standard, an analyzed ruby sphere with 0.44 mass fraction % Cr_2_O_3_ was used. X-ray data were collected with an HNU detector coupled to a 4 Pi Analysis digital beam control and data acquisition interface. During analysis, the beam was rastered over an area ≈100μm × 100 μm in size. Data was reduced using the conventional methods [25] with the aid of the DTSA software package [26].

#### 3.4.3 Mosaic Spread of the Ruby Spheres

One ruby was chosen randomly to analyze for the diffraction peak width using a double crystal diffractometer equipped with a Ge monochromator and analyzer that has an intrinsic crystal resolution of 0.005° (determined from the full width half maximum (FWHM) of a piece of float- zoned silicon). 2*θ*/*θ*-scans and rocking curves of the (300), (006), and (104) reflections of the rubies are recorded. The rocking curve scans measure the mosaic spread of the spheres, while the 2*θ*/*θ* scans show lattice strains.

#### 3.4.4 Guinier-Hägg Transmission Powder Technique

The Guinier-Hägg transmission technique was used as a second method for determining the lattice parameters. The principle of this technique was reported elsewhere, and details of this study will be reported separately. The Guinier-Hägg focussing x-ray powder camera [27] with the asymmetric geometry installed at the U.S. Geological Survey was manufactured by Incentive Research and Development AB (Stockholm, Sweden). It has been in continuous use until now, yielding x-ray powder data of high quality with trouble-free maintenance and utility and providing great sensitivity for recording weak lines. Prior to the measurements of the samples, the camera was aligned and calibrated. The powder pattern of SRM 640b was measured to check the cylindrical form and the adjustment of the camera. The measured cell parameter was 5.4307(3) Å, which agrees with the reported certified value of *a* = 5.43094(5) Å to within 2 standard deviations.

Five samples were prepared for this study. In each of five preparations, about 12 spheres (recovered from the original mounts) were crushed in a small agate mortar under toluene, but not ground. The powdered material (about 5 mg) was transferred to the planchet and mixed with silicon powder (NIST SRM 640b [28], untreated) as an internal standard. In all, about 60 spheres were consumed in the procedure. Samples are prepared by sticking the ruby powder onto a piece of Scotch “Magic Tape” placed over a 6 mm hole in a 21 mm diameter, thin aluminum plate or planchet. With the sample rotated, rather coarsely ground powder gives sharp, uniform diffraction lines on the film. The tape gives a weak, diffuse background, but no distinct lines on the film.

The 8 in × 1/2 in films used were Kodak Type SB5 and were measured with a NONIUS film viewer. In order to correct for the film shrinkage problem, Hägg et al. (1947) [29] has designed an automatic correction procedure. Before development the films are exposed in a special light box through a millimeter scale on a photographic glass plate (made by Zeiss), so that a scale graduated in tenth millimeters is recorded directly on the film parallel to the x-ray pattern. The film measurements were converted to Bragg angles (2*θ*) by multiplying the millimeter scale readings (after subtracting the measured zero reading), by a factor *k*, which is determined at six different angles by measurement of the Si lines. To test the trend of *k*, a sample composed of Si plus quartz was exposed, giving 6 Si lines and 27 quartz lines. Using the central 4 Si lines to standardize k, the quartz unit cell was determined by least-squares analysis of the determined 20 values. This led to unit cell parameters for quartz *a* = 4.9121(3) Å, *c* = 5.4033(6) Å, with the standard deviations for 2*θ* of 0.012°. These parameters were then used to calculate the expected 2*θ* values for quartz, and from these the corresponding *k* value for each line on the observed pattern. From these data it is clear that *k* remains constant within the standard error of measurement, except above 85°. Using this method, for each ruby sample *k* was determined by averaging *k* values obtained from the four central lines of Si. The resulting set of 2*θ* values for 9 to 11 lines of ruby was used to obtain the best unit cell parameters by using the least-squares program of Appleman and Evans [30]. This program automatically indexes at each cycle the input 2*θ* data (starting with an approximate unit cell) and yields a set of unit cell parameters (including cell volume) with their standard errors, the standard error of observed data of unit weight, and the variance-covariance matrix.

#### 3.4.5 Crystallographic Structural Parameters

Relevant information on the ruby spheres such as the structural parameters have been obtained using four ruby spheres and the Picker diffractometer at NRC, Canada. For this study, data was collected with the *θ*/2*θ* scan technique at a rate of 4°/min and profile analysis was applied. Graphite monochromatized MoKα radiation was used with the x-ray tube operated at 50 kV, 30 mA and 2*θ*_max_ = 120° for the ruby spheres and 90° for Al_2_O_3_. Five sets of intensity data were collected using the data collection routine DIFRAC [17].

### 3.5 Results

#### 3.5.1 Chromium Content of the Ruby Spheres

**Fig. 13:**
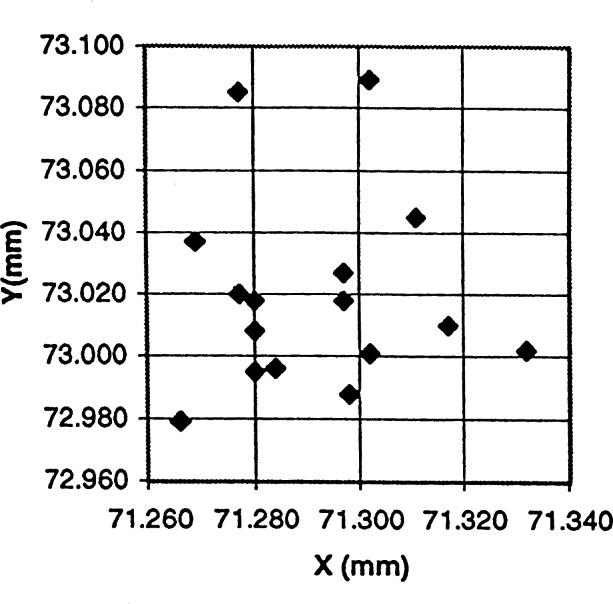
Analysis points of the secondary ruby sphere standard.

Using the electron microprobe technique, it was found that the measured value for Cr in the Smithsonian hypersthene was 0.53±0.02 (standard deviation) mass fraction % (compared to the nominal value of 0.51 %). The mean concentration and standard deviation of the 16 replicate measurements for Cr in the secondary standard (ruby pellet) was 0.44±0.04 mass fraction % (Table 3). The measurement precision of Cr (for both sample and secondary standard), based on the counting statistics is 0.02 mass fraction %. Considering the degree of surface relief and a small amount of charging that occurred on the ruby sample, the variation in measured Cr concentration is consistent with the sample being homogenous in composition. This sphere was found to have relatively large areas of microscopic surface roughness. The amount of variability of the measured concentration of the major Al in the sample is indicative of the surface roughness. Figure 13 shows the analysis points of the secondary ruby sphere standard.

**Fig. 14:**
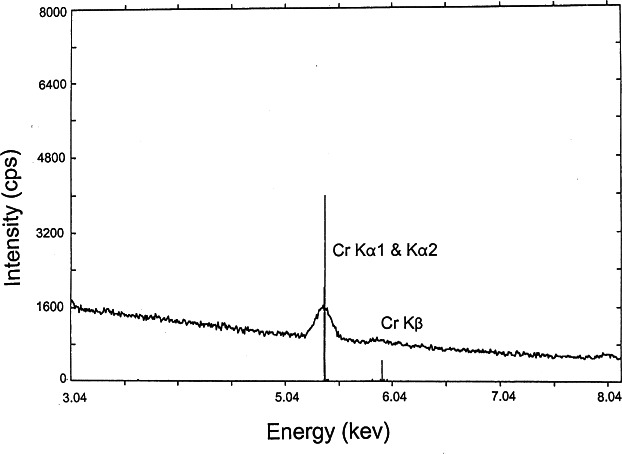
Energy dispersive spectrum showing the Cr Kα_1_, Kα_2_, and Kβ lines of a typical ruby sphere.

Table 4 shows the measured values of the Cr content of 15 spheres using the SEM/EDS method. Among them, sphere No. 15 is the secondary standard. The chromium composition was found to be relatively ho mogenous and was estimated to be 0.42 mole fraction %±0.011 % (expanded uncertainty). Figure 14 shows a typical EDS spectrum of one of the measured spheres. The peaks corresponding to Cr Kα_1_+Cr Kα_2_, and Cr Kβ are shown.

#### 3.5.2 Mosaic Spread of the Ruby Spheres

**Fig. 15:**
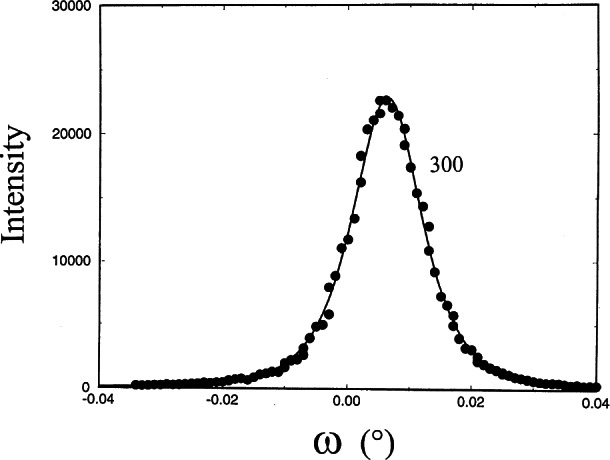
Rocking curves of a typical ruby crystal using a double crystal diffractometer. The reflection (300) is shown.

**Fig. 16:**
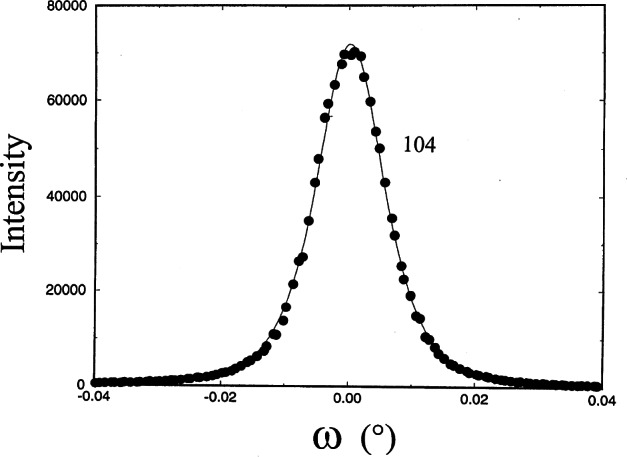
Rocking curves of a typical ruby crystal using a double crystal diffractometer. The reflection (104) is shown.

**Fig. 17:**
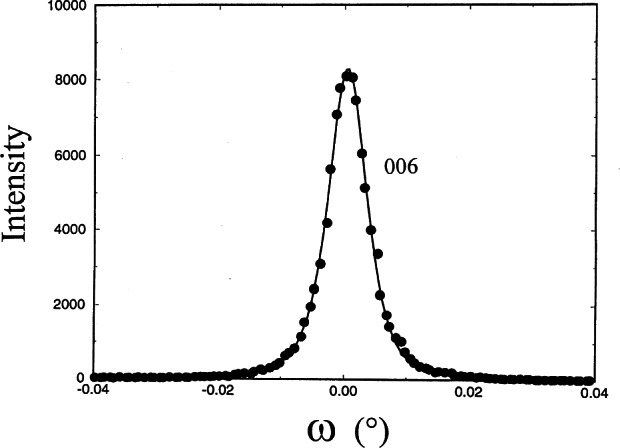
Rocking curves of a typical ruby crystal using a double crystal diffractometer. The reflection (006) is shown.

The x-ray rocking curves of the (006), (300) and (104) reflections were rather narrow, with FWHM from 0.007° to 0.012°, indicating a very small mosaic spread within the crystal. The variation of peak width indicates a small anisotropic mosaicity. The 2*θ*/*θ* scans of the (300), (104) and (006) reflections show narrow FWHMs of 0.027°, 0.0113°, and 0.0106°. From the Scherer formula [31], an average coherent crystalline size can be estimated, giving approximately 3600 Å for the distance perpendicular to the (300) and about twice this number, 8100 Å for distances perpendicular to (006). Therefore crystal growth of the ruby crystal appears to be anisotropic, with corresponding mosaicity due to the shape of these grains. Figures 15–17 show the rocking curves of reflections (300), (104) and (006) of a typical ruby sphere using a double crystal spectrometer. In general, negligible residual strain was found in the sample, which is indicative of the good quality of these spheres, fulfilling another requirement for being a good standard.

#### 3.5.3 Ruby Spheres as a SRM

Statistically, the lattice parameter measurements of the four groups of data (41 sets together) agree with each other within an acceptable range. The structural parameters of four ruby spheres determined using the Picker diffractometer are shown in Table 5. Small residual factors (*R*_F_ < 2 %, and *R*_W_ < 3 %) indicate correctness of the structure model and good quality of crystals. All atomic positions and thermal parameters are very close to each other, with a difference in general less than 2*σ*’s. For crystal C2-2, although the extinction and scale parameters differ somewhat, the other parameters are unaffected; the range of scale factors is consistent with the range of diameters. This result shows the corrections applied for the different measurements result in consistent structural parameters.

Before we present the result of lattice parameter determinations, various systematic errors that are associated with these measurements need to be addressed first.

##### 3.5.3.1 Systematic Error Corrections

The systematic corrections investigated include thermal expansion, absorption and eccentricity, horizontal divergence, vertical divergence, and refraction. Among them, only thermal expansion and refraction corrections were applied. Other corrections were too small to be considered.

Using the capabilities of single crystal diffractometer to bring any (*hkl*)-plane into the reflection position in more than just one setting, systematic errors due to zero-point offsets, sphere of confusion, sample displacements and absorption can be minimized. Such procedures depend on the diffractometer geometry and have been described elsewhere [3].

###### Thermal effect

Among all factors affecting the lattice parameters of the rubies, thermal expansion appears to have the most significant effect. Because of the small amount of Cr, the thermal expansion coefficient of ruby was assumed to be the same as Al_2_O_3_. The anisotropic thermal expansion coefficient of Al_2_O_3_ was reported by Campbell and Grain [9], and was more recently reviewed by Munro [32]. The thermal expansion coefficient curve is quadratic in nature (up to 1800 °C). For data near the room temperature range, it can be assumed to be linear. Within the range of 0 °C to 100 °C the *a* and *c* axes of α-Al_2_O_3_ were found to expand linearly but anisotropically [32]. Under these conditions, the corrections can be calculated according to the following expression:

$$\begin{array}{l} a^{\prime }(25{}^{\circ}C)=a[1+\alpha_{a}(T-25)],\;\text{where}\;\alpha_{a}=5.0\times 10^{-6}\\ c^{\prime }(25{}^{\circ}C)=c[1+\alpha_{c}(T-25)],\;\text{where}\;\alpha_{c}=6.66\times 10^{-6} \end{array}$$

[8]The data collection of these experiments took place in a range of 19 °C to 26 °C. Based on the above equations, corrections to 25 °C have been applied.

###### Refraction

There are two contributions to the refraction correction [10]. One corresponds to the Snell’s law correction: δ*d/d* = −*v* cot*θ* = (1−*n*) cot*θ*. This part of correction is too small to be included. The other part, refraction due to change of wavelength is 1−*n* = (2.71 × 10^−6^) *ρλ*^2^ (ΣZ/Σ*a*). In this equation, *ρ* is the density (taken as 3.98 g/cm^3^ [7], *λ* is the wavelength, *Z* is the atomic number and *a* is the atomic weight. In the expression δ*d/d* = (1−*n*)/*n*, (1−*n*) = 2.69×10^−6^ for Mo radiation, and (1−*n*) = 1.27×10^−5^ for Cu radiation.

###### Absorption

According to Hubbard and Mauer [10], the absorption correction for the Si crystal that they studied is about the value of 0.000079 Å in 5.43 Å, or 15×10^−6^ for Si at 2*θ* of 80°. An estimation shows that this value will be much smaller in the case of the ruby due to the following two reasons. (1) The Si spheres were 0.25 mm in diameter, whereas the rubies are only about 0.15 mm, therefore the volume of the rubies are about 0.14 that for Si, and (2) for Cu radiation, the *μ* value is 141 cm^−1^ for Si, but 124 cm^−1^ for ruby. This indicates that in the *θ* range of 0° to 80°, the transmission factor ranges from approximately 0.1 to 0.2 for Si, and from 0.32 to 0.39 for Al_2_O_3_. Absorption was estimated to affect the lattice parameters of the ruby in the order of ≈10^−5^ using Cu radiation, and negligible for Mo radiation. Accurate absorption correction is a non-trivial process. But because of the small magnitude, the absorption correction was not applied.

###### Divergence of x-ray beams

In the equation δ*d/d* = (−2cos*θ*/*λ*) *δθ* = *A_v_*^2^/6 [10], since the collimator used has a small aperture, the A_v_ value is of an approximate value of 0.05°, and the δ*d* value is ≈10^−6^ Å. The axial divergence is a negligible source of error compared with the other causes. These values are too small in magnitude to be measured.

##### 3.5.3.2 Lattice Parameter Determinations

A total of 45 sets of 4-circle diffractometer data were measured and analyzed. Measurement results are listed in Table 6. There are a total of four subsets of data. Consistency of diffractometer measurements were performed by cross comparison of data measured with these diffractometers using the same spheres. Satisfactory data were obtained for these comparisons (sample pairs of 2a,2b; 5a,5b; 6a,6b; 29a,29b; L1–17, L2–17; L1–22, L2–22). The first set was measured using CuKα radiation at the NRC of Canada on a CAD4 diffractometer (C1). The second set of data was also measured at NRC Canada using Mo Kα radiation, but with a Picker diffractometer (C2). The third subset was measured at Bell Laboratories with Mo radiation (L2), and the fourth one was also measured at Bell Laboratories using Cu radiation (L1).

##### 3.5.3.3 Statistical Analysis of the Lattice Parameter Results

The certified value and its uncertainty represents a consensus of the four sets of measurements. Each of the four sets of measurements for a particular lattice parameter (*a* and *c*) is reduced to a mean value. The certified value for the lattice parameter is the mean of the resulting four mean values. The standard uncertainty of the certified value is the standard error of the mean, which is equal to the sample deviation of the four mean values divided by the square root of 4. The associated degree of freedom is 3 and the corresponding coverage factor is equal to the standard uncertainty times the coverage factor. The interval contains the lattice value with 95 % level of confidence. Readers are to refer to reference [37] for the expression of uncertainty in measurement for details on the procedure and terminology.

The certified value of the lattice parameters (mean value) are *a* = 4.76080 ± 0.00029 Å (expanded uncertanties), and *c* = 12.99568 ± 0.00087 Å. The interval defined by the estimate the expanded uncertainty provides an interval with an approximate 95 % level of confidence. It is equal to the standard uncertainty times a coverage factor, *k* (*k* = 3.18). The standard uncertainty represents the standard deviation of the estimate. The coverage factor k accounts for the degrees of freedom in the estimation of the uncertainty. These values fall well within the results obtained from the international round-robin study (*a* = 4.7608 Å ± 0.0062 Å [expanded uncertainties], *c* = 12.9979 Å ± 0.020 Å). However, the expanded uncertainties are significantly smaller in our certified values. Therefore the round-robin data can not be used as certified data, only as a comparison. The five significant digits reported for these certified lattice parameters is more precise than the four obtained in typical laboratories. This batch of ruby spheres therefore will serve well as a diffractometer alignment standard.

**Fig. 18:**
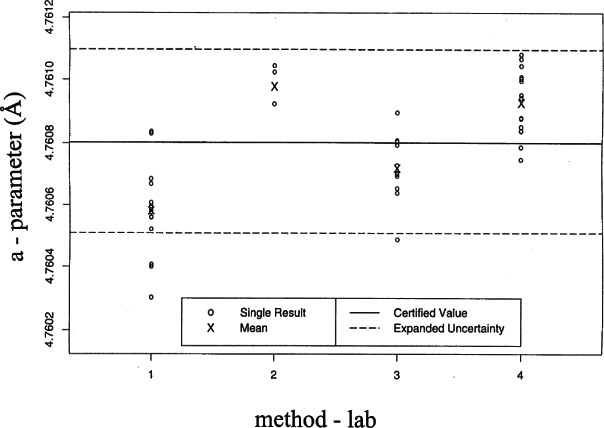
The four sets of measurements, the certified value, and the expanded uncertainty for the lattice parameters *a* using single crystal diffractometers. The expanded uncertainty interval contains the means of the four sets of measurements.

**Fig. 19:**
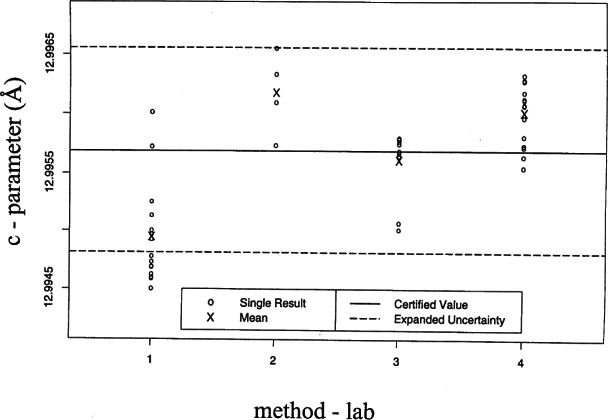
The four sets of measurements, the certified value, and the expanded uncertainty for the lattice parameters *c* using single crystal diffractometers. The expanded uncertainty interval contains the means of the four sets of measurements.

Figures 18 and 19 display the four sets of measurements, the certified value, and the expanded uncertainty for the lattice parameters *a* and *c*, respectively. For both lattice parameters, the expanded uncertainty interval contains the means of the four sets of measurements.

The measurement procedure described above strives to compensate for differences between diffractometer. However, total elimination of hardware influences cannot be achieved under any circumstances. Examination of the data from the similar ENRAF-NONIUS CAD-4 diffractometers shows a small residual offset of the unit cell parameters measured, even though a similar procedure using the same high-level software was used. Since the control over the two experiments in different locations was not absolute, small differences are expected. We can speculate on the influence of some differences: for instance, the diffractometers have slight differences in their respective low-level positioning and automation system, affecting the positioning feedback system. The quality of the x-ray optical elements and their alignment will also influence the peak profiles of the diffracted intensities. Environmental variables such as air temperature and air pressure are also going to affect the results, even though care was taken to minimize their influences. However, the temperature variamtions in the rooms and in radiation safety enclosures could not be fully taken into account due to varying conditions during the measurements and physical distance between the diffractometers used.

A comparison of the ruby cell parameters with the alumina data [33-35] indicates that the rubies have larger lattice constants. The increase in cell volume of the ruby spheres over that for pure corundum is expected since the effective ionic radius of Al^3+^ (0.535 Å) is smaller than that of Cr^3+^(0.615 Å) [36]. Table 7 shows reported cell parameters for alumina. The lattice parameters of the alumina from Ref. [18] have a small error associated because there was only one sample.

#### 3.5.4 Guinier-Hägg Transmission Camera Data

The Guinier-Hägg powder data of the five ruby samples show good agreement with the single crystal data, and thus confirm the certified lattice parameters are free from systematic errors (Table 8). After thermal expansion and refraction corrections were applied (discussed below), the mean values of these lattice parameters are *a* = 4.7610 ± 0.0013 Å (expanded uncertainty), and *c* = 12.9954 ± 0.0034 Å.

Various possible errors involved with the Guinier-Hägg technique that may affect the accuracy of cell parameters include errors due to x-ray beam divergence, film shrinkage and thermal expansion. Other problems caused by specimen displacement, transparency effect, and absorption can mostly be compensated by using the internal standard, Si 640b [28].

##### Thermal Expansion Effect

The same corrections as those for the single crystal diffractometer data were applied here. The corrections were calculated according to the following expression:

$$\begin{array}{l} a^{\prime }\left(25{}^{\circ}C\right)=a\left[1+\alpha_{a}\left(25-T\right)\right],\text{where}\;\alpha_{a}=5.0\times 10^{-6}\\ c^{\prime }\left(25{}^{\circ}C\right)=c\left[1+\alpha_{c}\left(25-T\right)\right],\text{where}\;\alpha_{c}=6.66\times 10^{-6} \end{array}$$

[8]The data collection of these experiments took place at 21°C. Based on the above equations, a correction of 4° (21° to 25°C) was applied.

##### Refraction

Similar corrections as that for the single crystal diffractometers was applied here. In the expression δ*d/d* = (1−*n*)/*n*, (1−*n*) = 2.69 × 10^−6^ for Mo radiation, and (1−*n*) = 1.27 × 10^−5^ for Cu radiation. The corrections are also of relatively small quantity.

##### Absorption

The absorption coefficient of Al_2_O_3_ (124 cm^−1^) and Si (141 cm^−1^) is of similar order of magnitude, and the difference between the Cr-doped Al_2_O_3_ is expected to be < 17 cm^−1^. Therefore the difference in the *d*-spacing aberration of the rubies due to absorption can be largely compensated for by mixing in with the Si standard, 640b [28].

##### Sample Displacement

A condition for the camera to give sharp interference lines on a film is that the powder sample is situated on the cylinder defined by the film. Since in the actual procedure, the sample is adhered to a piece of thin scotch tape which is in turn attached to a flat metal ring rotated in a plane tangent to the focusing cylinder, a minor deviation of the sample from the correct location causes a slight broadening of the interference lines and displacements of their positions on the film. This problem can again be largely corrected for by mixing the sample with the Si standard [28].

##### Sample Transparency

The extraordinary focusing property of the Guinier-Hägg transmission camera also eliminates to a large extent the influence of the thickness of the specimen. As pointed out by Jenkins and Snyder [38], this error can be corrected for by intimate mixing with Si as an internal standard [28].

##### Divergence of the X-ray Beam

Axial divergence is expected to be a negligible source of error compared with the other causes. Klug and Alexander [31] have shown that because of the axial divergence of the beam, the center of the blackening of a line in a powder camera will be shifted by a small angular amount, δ*θ*= −[(1+*x*)/96] (*H*/*R*)^2^ cot 2*θ*, where *R* is the radius of the film, *H* is the axial divergence of the beam in a camera of radius *R*. If the beam is homogenous, *x* = 0. Since the collimator used has a small aperture, the *H*/R value is small and the correction is trivial. For example, with a *H*/*R* value of 1/50, a δ*θ* of 0.0004° is obtained at 2*θ* at 30°, and a δ*θ* of 0.00004° is obtained and at 80° 2*θ*. These values are much too small in magnitude to be measured, and can be ignored.

### 3.6 Discussion

In recent years, due to the advances of computer and x-ray diffractometry technology, x-ray diffractionists have a great number of venues of obtaining data with adequate accuracy. In order to achieve high accuracy in the range of 0.001 % to 0.0001 %, extra effort is needed. In the following, a list of factors that may affect the accuracy of lattice parameter determination using a single crystal diffractometer is given.

Based on the results of the round-robin project and the current cell parameter certification project, the use of SRM 1990 is expected to enhance the alignment capability of single crystal x-ray diffractometers in industrial, government and academic x-ray laboratories. The recommended use of the SRM will also be discussed.

#### 3.6.1 Factors Affecting Accuracy of Single-crystal Diffractometer Alignment and Lattice Parameter Determination (Serial Diffractometers Only)

1. The precision of diffractometer gears, spindle pitch, encoders, etc. are important for achieving accurate measurements, and diffractometers must be carefully adjusted to avoid mechanical problems.
2. The goniostat must be well aligned, with a sphere of confusion as small as possible. Typical values for a modern diffractometer are of the order of 10 μm to 20 μm. This is the maximum wobble of the sample when it is rotated about all the axes (When its reflections are aligned in the scattering plane). For a *κ*-axis system, the sphere of confusion indicates how close the four axes intersect in one point. For an Eüler system, the sphere of confusion describes how close to a circle the Eüler cradle is, and how well the three remaining axes intersect in one point. Usually the *κ*-axis system tends to have a smaller sphere of confusion. Calibration constants are supplied with the goniometer and the aperture system, and should be checked carefully.
3. The major task of the alignment procedure is to direct the primary x-ray beam through the center of the goniometer and through the center of the receiving aperture when positioned at *θ* = 0°. The beam must be carefully centered. The routine to center a reflection is important. It is recommended from the international round-robin result that the alignment routine should incorporate the King and Finger’s algorithm [3, 4, 39] for calculating various alignment corrections such as offsets of detector, x-ray tube in both horizontal and vertical direction, the angular offsets of the monochromator, and the offsets of the crystals from the center of the primary beam, *X,Y*, and *Z* direction.
4. Reflections with high angles are important to achieve accuracy. As in the Bragg equation, δ*d* = (−*n*/2)(cot*θ*/sin*θ*)δ*θ*, it can be seen that the error in δ*d* is relatively small when higher angle reflections are used. In the case of the ruby spheres, there are a great number of strong reflections at high angles, therefore the usual concerns of disadvantages of back reflections, namely, low intensity, lower peak-to-background ratio and broadening by wavelength dispersion will not be a problem here. Table 9 lists the high angle reflections that can be used for initial orientation, and for accurate alignment of the SRM.
5. Most diffractometer programs assume the intensity ratio in the α_1_/α_2_ doublet to be 2:1. This is true only if the diffractometer optical system is achromatic (slit/collimators). If a monochromator is used, then a deviation from the 2:1 intensity is likely [40]. Applying the wrong ratio will therefore give an incorrect value for the position.
6. The profile shape of reflections chosen has a strong influence on the accuracy of angle measurement. Therefore a profile analysis routine is critical for determining the correct peak positions. Different software may use different methods, i.e., centroid of the peak, full-width-half-maximum, and mid-chord method, etc. The profile-fitting program must be chosen and used carefully.
7. If the routine of eight-reflection is not used, then systematic errors such as the 2*θ*_0_ correction should be applied for obtaining accurate 2*θ* values.
8. Different computer programs that perform least-squares lattice parameter refinements may use different routines. One should understand these routines and how the associated errors are calculated. This is important for inter-laboratory comparison of lattice parameters.

#### 3.6.2 Recommended Use of SRM 1990

In order to obtain reliable data, it is recommended that users perform regular alignment checks of the diffractometers using the SRM ruby spheres. The spheres should be mounted using a minimal amount of adhesive material and on the center of the tip of a fiber or preferably a capillary with a diameter slightly smaller than that of the spheres (i.e., <0.1 mm). The crystal should be kept mounted permanently on a goniometer head (i.e., devote one goniometer head to that purpose). The orientation matrix should be known and the results of alignment should be kept and used for comparison later. A complete set of intensity data should be collected and the structure refinement results should yield a low residual value equivalent to those in Table 5.

It is easy to center the ruby sphere so that the misalignment is minimal. The center of the ruby sphere then defines the center of the diffractometer. Maximizing the intensity of a given reflection ensures that the highest intensity part of the beam intercepts the ruby sphere.

During the alignment process, high angle reflections should be used. Relatively low angle reflections such as 006, 0012, and 300 can be used for initial orientation of the spheres [(Table 9, part (A)]. Further accurate alignment can be carried out with the reflections as indicated in Table 9 [part (B)]. In this table, the Miller indices, multiplicity of the reflection, the calculated structure amplitudes, *Fc*, and the 2*θ* (CuKα_1_) and 2*θ* (MoKα_1_) are listed. The 2*θ* and *Fc* values were calculated based on the certified lattice parameters and structure of sample C1-1. The *Fc* values are not certified, but are for reference purposes.

In addition to being excellent standards for alignment of conventional diffractometers, these ruby spheres will also be a valuable standard for instrument calibration for diffractometers equipped with CCD detectors. CCDs and other area detectors are a recent addition to the field of crystallography. With these instruments, the diffracted intensities are measured over a large solid angle. The diffracted plane then varies and is no longer well defined as is the case for a serial diffractometer. For an achromatic system, the doublet splitting is symmetrical with respect to the center of the diffractometer. If an incident beam monochromator is used, then the system is asymmetrical, giving sharp reflection on one side where the dispersion is compensated (non-dispersive side), while giving broad reflections the opposite side (dispersive side). For a calibration of the flat CCD, the distance of the detector to the sample has to be determined accurately, then the flat field is unwarped and mapped onto a spherical detector. This process will benefit from an accurate lattice parameters standard, since both the distance, the beam offsets, roll and yaw of the detector can be determined using the ruby standard.

## 4. Summary

X-ray structural determinations using automatic data collection and structure solution schemes require accurate initial cell parameter data. Until now, no certified standard was available for the evaluation of the diffractometer condition, alignment and inter-laboratory comparison of data. The result of this work is expected to enhance the alignment capability of single crystal x-ray diffractometers in industrial, government and academic x-ray laboratories. Therefore the success of this project will have a significant impact on accurate scientific investigations using single crystal diffractometers.

The lattice parameter is being certified as *a* = 4.76080 ± 0.00029 Å (expanded uncertainty), and *c* = 12.99568 ± 0.00087 Å. Five different samples of powdered rubies were measured on a Guinier-Hägg transmission camera. The values of *a*=4.7610 ± 0.0013 Å (expanded uncertainty), and *c* = 12.9954 ± 0.0034 Å give good agreement with the values obtained from the single crystal spheres. Among all systematic errors, only the thermal acorrection and refraction corrections were applied. the auxiliary data on the Cr-content will also be useful for microanalytical calibrations.

## Figures and Tables

**Table 1:** Lattice parameters of the ruby spheres after the application of the thermal and refraction corrections. Identification of each laboratory is not given. The total number of experiments are greater than the number of the laboratory because of multiple experiments performed in some laboratories

Lab No.	Expt. No.	Rad. Ty.	Diff. Ty.	a (Å)	b (Å)	c (Å)	*T* (°C)	Ave. (each laboratory)		
								*a*(Å)	*b*(Å)	*c*(Å)
1	1	Mo	CAD4	4.7569	4.7574	12.9848	25.00			
2	2	Mo	AFC6S	4.7593	4.7595	13.0018	−145.00			
3	3	Cu	CAD4	4.7573	4.7622	12.9942	21.00			
4	4	Mo	CAD4	4.7595	4.7595	12.9873	23.00	4.7601	4.7601	12.9899
	5	Mo	CAD4	4.7608	4.7608	12.9924	23.00			
5	6	Mo	CAD4	4.7612	4.7612	12.9960	25.00	4.7612	4.7612	12.9985
	7	Mo	CAD4	4.7612	4.7612	13.0010	25.00			
6	8	Mo	CAD4	4.7599	4.7599	12.9924	25.00	4.7608	4.7608	12.9957
	9	Mo	CAD4	4.7618	4.7618	12.9989	−145.0			
7	10	Mo	P3	4.7597	4.7591	12.9915	22.00			
8	11	Mo	CAD4(1)	4.7601	4.7601	12.9950	20.00	4.7617	4.7617	12.9974
	12	Mo	CAD4(2)	4.7602	4.7602	12.9946	20.00			
	13	Cu	CAD4(3)	4.7616	4.7616	12.9956	20.00			
	14	Mo	CAD4(4)	4.7599	4.7599	12.9924	20.00			
	15	Mo	SMART	4.7640	4.7668	13.0095	20.00			
9	16	Mo	CAD4	4.7609	4.7609	12.9951	20.00			
10	17	Mo	R3m	4.7619	4.7619	12.9925	23.00			
11	18	Mo	P3	4.7658	4.7661	13.0097	25.00	4.7623	4.7622	12.9991
	19	Cu	P3	4.7589	4.7584	12.9885	25.00			
12	20	Mo	CAD4	4.7606	4.7614	12.9965	23.00			
13	21	Mo	AFC6	4.7642	4.7637	13.0067	23.00			
14	22	Mo	AFC6	4.7654	4.7647	13.0161	26.00			
15	23	Mo	Huber	4.7696	4.7696	13.0207	20.00			
16	24	Cu	CAD4	4.7603	4.7595	12.9959	20.00			
17	25	Ag	CAD4	4.7603	4.7610	12.9947	20.00			
18	26	Cu	CAD4	4.7588	4.7588	12.9926	20.00			
19	27	Mo	Kuma K	4.7602	4.7602	12.9925	22.00			
20	28	Mo	Stoe AED	4.7614	4.7614	12.9952	24.00	4.7623	4.7622	12.9991
	29	Mo	Stoe AED	4.7621	4.7621	12.9977	24.00			
	30	Mo	P4	4.7634	4.7634	13.0004	24.00			
	31	Mo	P4	4.7629	4.7629	13.0024	24.00			
21	32	Mo	CAD4	4.7594	4.7577	12.9755	20.00	4.7586	4.7595	12.9881
	33	Mo	CAD4	4.7606	4.7605	12.9905	20.00			
	34	Mo	CAD4	4.7557	4.7602	12.9985	20.00			
22	35	Mo	CAD4	4.7559	4.7548	12.9843	23.00			
23	36	Mo	CAD4	4.7592	4.7596	12.9932	23.00			
24	37	Mo	P21	4.7662	4.7642	13.0032	25.00			
25	38	Cu	Russia4C	4.7638	4.7639	13.0062	25.00			
26	39	Mo	CAD4	4.7555	4.7558	12.9870	25.00			
27	40	Mo	Stoe 4C	4.7614	4.7614	12.9983	22.00			
28	41	Mo	AED 2	4.7639	4.7639	13.0293	22.00			
29	42	Mo	CAD4	4.7621	4.7621	12.9983	22.00			
30	43	Cu	CAD4	4.7585	4.7588	12.9933	20.00			
31	44	Mo	CAD4	4.7609	4.7605	12.9945	25.00			
32	45	Mo	Rigaku	4.7570	4.7570	13.0020	25.00			

**Table 2:** Round-Robin Results of the reference zeolite crystals, Al_2_Si_34_O_72_ **·** 2(C_5_H_5_O) **·** 2HF. The identification of each laboratory is not given. The total number of experiments are greater than the number of the laboratory because of multiple experiments performed in some laboratories.

Lab No.	Expt. No.	Rad. Ty.	Diff. Ty.	a (Å)	b (Å)	c (Å)	*T*(°C)	Ave. (each laboratory)		
								*a*(Å)	*b*(Å)	*c*(Å)
1	1	Mo	CAD4	18.8286	14.0963	7.4316	25.20			
2	2	Mo	AFC6S	18.7568	14.0915	7.4125	−145.00			
3	3	Cu	CAD4	18.8320	14.1060	7.4345	21.00			
4	4	Mo	CAD4	18.8309	14.1016	7.4362	23.00	18.8306	14.1014	7.4353
	5	Mo	CAD4	18.8303	14.1012	7.4344	23.00			
5	6	Mo	CAD4	18.8360	14.1060	7.4340	25.00	18.8356	14.1055	7.4350
	7	Mo	CAD4	18.8352	14.1050	7.4360	25.00			
6	8	Mo	CAD4	18.8257	14.1014	7.4323	25.00			
7	9	Mo	P3	18.8270	14.1030	7.4300	22.00			
8	10	Mo	CAD4(1)	18.8326	14.0977	7.4341	20.00	18.8290	14.1023	7.4341
	11	Mo	CAD4(2)	18.8265	14.1020	7.4333	20.00			
	12	Mo	CAD4(3)	18.8318	14.1032	7.4351	20.00			
	13	Mo	CAD(4)	18.8250	14.1073	7.4340	20.00			
9	14	Mo	CAD4	18.8249	14.1037	7.4361	20.00			
10	15	Mo	R3m	18.8410	14.1140	7.4360	23.00			
11	16	Mo	P3	18.8500	14.1095	7.4910	25.00	18.8350	14.1006	7.4606
	17	Cu	P3	18.8199	14.0918	7.4303	25.00			
12	18	Mo	CAD4	18.8300	14.1060	7.4329	22.00			
13	19	Mo	AFC6	18.8503	14.1459	7.4400	23.00			
14	20	Mo	AFC6	18.8632	14.1284	7.4501	26.00			
15	21	Mo	Huber	18.8500	14.1160	7.4420	20.00			
16	22	Cu	CAD4	18.8348	14.1019	7.4350	20.00			
18	23	Cu	CAD4	18.8343	14.0924	7.4305	20.00			
19	24	Mo	Kuma K	18.8336	14.0979	7.4344	22.00			
20	25	Mo	Stoe AED	18.8394	14.1118	7.4395	24.00	18.8404	14.1028	7.4381
	26	Mo	Stoe AED	18.8332	14.1192	7.4398	24.00			
	27	Mo	P4	18.8442	14.0905	7.4352	24.00			
	28	Mo	P4	18.8450	14.0896	7.4378	24.00			
21	29	Mo	CAD4	18.8050	14.0800	7.4240	20.00	18.8070	14.0887	7.4280
	30	Mo	CAD4	18.8150	14.0870	7.4370	20.00			
	31	Mo	CAD4	18.8010	14.0990	7.4230	20.00			
22	32	Mo	CAD4	18.8210	14.0940	7.4313	23.00			
23	33	Mo	CAD4	18.8000	14.0900	7.4270	23.00			
24	34	Mo	P21	18.8513	14.0757	7.4407	25.00			
25	35	Cu	Russia4C	18.8450	14.1050	7.4387	25.00			
26	36	Mo	CAD4	18.8220	14.0950	7.4340	25.00			
27	37	Mo	Stoe 4C	18.8400	14.1050	7.4390	22.00			
28	38	Mo	AED 2	18.8609	14.1283	7.4507	22.00			
29	39	Mo	CAD4	18.8430	14.0981	7.4383	22.00			
30	40	Cu	CAD4	18.8269	14.0758	7.4303	22.00			
31	41	Mo	CAD4	18.8235	14.1027	7.4339	25.00			
32	42	Mo	Rigaku	18.8280	14.1230	7.4380	25.00			

**Table 3:** Results of analysis points on the standard sphere

No.	Mass fraction, %		Mole fraction, %	
	Al	Cr	Al	Cr
1	50.95	0.51	99.48	0.52
2	51.28	0.43	99.57	0.43
3	51.09	0.52	99.47	0.53
4	52.11	0.49	99.51	0.49
5	50.71	0.38	99.61	0.39
6	51.72	0.41	99.59	0.41
7	51.39	0.40	99.60	0.40
8	52.25	0.41	99.59	0.41
9	51.27	0.42	99.58	0.42
10	51.48	0.44	99.56	0.44
11	51.53	0.44	99.56	0.44
12	51.78	0.45	99.55	0.45
13	51.88	0.43	99.57	0.43
14	51.62	0.43	99.57	0.43
15	54.26	0.48	99.54	0.46
16	51.05	0.40	99.60	0.40

Average	51.65	0.44	99.56	0.44
Standard dev.	0.41	0.04	0.41	0.04

**Table 4:** Chromium content of the ruby spheres (mole fraction, %)

No.	Al	Cr
1	99.59	0.41
2	99.58	0.42
3	99.55	0.45
4	99.60	0.40
5	99.57	0.43
6	99.59	0.41
7	99.55	0.45
8	99.58	0.42
9	99.61	0.39
10	99.57	0.43
11	99.56	0.44
12	99.59	0.41
13	99.60	0.40
14	99.60	0.40^a^
15	99.56	0.44

Average		0.42
Standard deviation		0.019
Expanded uncertainty		0.011

a Secondary standard.

**Table 5:** Structural parameters for four ruby spheres and one alumina crystal (auxiliary information, not certified values), measured with a Picker diffractometer at NRC Canada using Mo radiation (space group, $R\bar{3}c$.)

Sphere No.		C2-1	C2-2	C2-3	C2-4	Al_2_O_3_
No. refls. measured		880	886	1039	942	2844
No. observed/unique		414/446	415/446	414/446	410/446	237/242
Al	*z*	0.35227(2)	0.35229(3)	0.35225(2)	0.35226(2)	0.35129(1)
	u11	0.00312(8)	0.00309(9)	0.00306(8)	0.00300(9)	0.00338(5)
	u13	0.00355(10)	0.00361(11)	0.00343(10)	0.00349(11)	0.00335(5)
O	*x*	0.69374(11)	0.69373(13	0.69380(13)	0.69382(12)	0.69367(5)
	u11	0.00369(12)	0.00364(14)	0.00362(12)	0.00356(13)	0.00359(6)
	u33	0.00369(13)	0.00364(15)	0.00352(13)	0.00366(14)	0.00394(8)
	u12	0.00173(13)	0.00166(16)	0.00170(14)	0.00164(15)	0.00166(7)
	u13	0.00029(6)	0.00030(7)	0.00029(6)	0.00029(7)	0.00035(3)
Scale		0.2673(1)	0.2472(1)	0.2820(1)	0.2792(1)	0.1496(4)
Ext. (μm)		0.76(3)	0.32(2)	0.75(3)	0.66(3)	0.52(3)
*R*_F_		0.0158	0.0178	0.0152	0.0162	0.0134
*R*_w_		0.0257	0.0318	0.0262	0.0284	0.0068

**Table 6:** Lattice parameters for 39 ruby spheres (SRM 1990). A total of 45 measurements were performed using four units of single crystal diffractometers (C1: Enraf-Nonius at NRC Canada, C2: Picker at NRC Canada, L1 and L2: Enraf-Nonius at Lucent Technologies), after applying both thermal expansion and refraction corrections

No.	ID	Rad Type	*a* (Å)	*c* (Å)	*T* (°C)
1	C1-1	Cu	4.760686(51)	12.99513(15)	20.4
2	C1-2a	Cu	4.760303(65)	12.99450(17)	20.2
3	C1-2b	Cu	4.760400(54)	12.99462(14)	20.3
4	C1-3	Cu	4.760597(63)	12.99478(17)	20.7
5	C1-4	Cu	4.760561(51)	12.99500(14)	20.2
6	C1-5a	Cu	4.760584(67)	12.99468(19)	22.6
7	C1-5b	Cu	4.760577(60)	12.99492(17)	22.6
8	C1-6a	Cu	4.760836(58)	12.99572(16)	19.5
9	C1-6b	Cu	4.760830(67)	12.99601(19)	21.0
10	C1-7	Cu	4.760592(58)	12.99525(16)	20.4
11	C1-8	Cu	4.760521(59)	12.99469(16)	19.2
12	C1-9	Cu	4.760559(54)	12.99496(15)	19.0
13	C1-10	Cu	4.760608(63)	12.99473(17)	20.0
14	C1-11	Cu	4.760669(74)	12.99459(23)	19.9
15	C1-12	Cu	4.760406(62)	12.99460(14)	19.9
16	C2-1	Mo	4.760924(30)	12.99609(16)	26.2
17	C2-2	Mo	4.761024(60)	12.99633(16)	26.2
18	C2-3	Mo	4.760924(60)	12.99573(16)	26.2
19	C2-4	Mo	4.761044(60)	12.99655(14)	26.2
20	L2-1	Mo	4.760795(70)	12.99575(20)	20.6
21	L2-3	Mo	4.760705(59)	12.99567(23)	20.6
22	L2-17	Mo	4.760809(62)	12.99578(17)	19.4
23	L2-18	Mo	4.760805(62)	12.99578(16)	19.4
24	L2-22	Mo	4.760695(79)	12.99576(27)	19.3
25	L2-24	Mo	4.760655(66)	12.99568(18)	19.3
26	L2-25	Mo	4.760897(83)	12.99507(30)	19.0
27	L2-27	Mo	4.760487(80)	12.99501(16)	19.0
28	L2-28	Mo	4.760640(98)	12.99579(20)	19.2
29	L2-29a	Mo	4.760700(81)	12.99564(17)	19.2
30	L2-29b	Mo	4.760730(80)	12.99574(17)	19.2
31	L1-2	Cu	4.760881(98)	12.99597(26)	25.8
32	L1-4	Cu	4.761081(94)	12.99629(28)	26.0
33	L1-5	Cu	4.760788(75)	12.99572(20)	25.8
34	L1-7	Cu	4.761045(71)	12.99629(20)	25.8
35	L1-8	Cu	4.760853(75)	12.99581(21)	25.8
36	L1-9	Cu	4.761005(84)	12.99608(22)	25.8
37	L1-10	Cu	4.760944(67)	12.99628(18)	25.8
38	L1-11	Cu	4.760879(80)	12.99607(22)	22.3
39	L1-12	Cu	4.760953(79)	12.99618(21)	22.5
40	L1-14	Cu	4.761009(82)	12.99613(22)	25.8
41	L1-15	Cu	4.760749(110)	12.99564(32)	22.2
42	L1-16	Cu	4.760839(65)	12.99573(19)	22.5
43	L1-17	Cu	4.761067(84)	12.99633(18)	25.8
44	L1-22	Cu	4.760841(64)	12.99555(18)	25.8
45	L1-23	Cu	4.760998(112)	12.99612(30)	25.8

**Table 7:** Literature lattice parameters of alumina (and of rubies for comparison)

Material	Reference	*a* (Å)	*c* (Å)
Alumina	Ishizawa et al. [33]	4.754(1)	12.99(2)
Alumina	Morris, et al. [34]	4.7588(1)	12.992(1)
Alumina	Newnham et al. [35]	4.7589	12.991
Alumina	This work [18]	4.75999(3)	12.99481(7)
SRM rubies	This work	4.76080(29)	12.99568(87)
SRM rubies	This work (Guinier-Hägg)	4.76093(31)	12.9959(23)

**Table 8:** Crystallographic Data for the ruby spheres using the Guinier-Hägg camera technique, data taken at 22 °C

			Sample No.		
	I	II	III	IV	V
*a* (Å)	4.76091(47)	4.76073(20)	4.76117(48)	4.76053(67)	4.76065(44)
*c* (Å)	12.9977(14)	12.99577(53)	12.9967(13)	12.9937(16)	12.9934(15)
*V* (Å^3^)	255.14(4)	255.08(5)	255.15(6)	255.02(7)	255.03(5)

Average with thermal and refraction correction:

*a* = 4.7610 ± 0.00013 Å (expanded uncertainty)

*c* = 12.9954 ± 0.0034 Å (expanded uncertainty)

**Table 9:** High-angle 2*θ* reflections for obtaining both initial orientation of the spheres and for accurate alignment of diffractometers. *M* is the multiplicity, the *h k l* values are the Miller indices and *Fc* is the calculated structure amplitude.

*h*	*k*	*l*	*M*	*Fc*	2 *θ* (CuKα_1_) (°)	2*θ*(MoKα_1_) (°)
(A) For initial orientation						
0	1	2	6	47	25.567	11.694
1	0	4	6	83	35.139	15.978
1	1	0	6	61	37.762	17.137
0	0	6	2	13	41.665	18.848
3	0	0	6	141	68.180	29.910
0	0	12	2	57	90.678	38.232
(B) For accurate alignment						
1	3	4	12	51	91.144	
2	2	6	12	74	95.203	
0	4	2	6	38	98.342	
2	1	10	12	64	101.031	
4	0	4	6	38	103.262	
3	1	8	12	36	110.931	
2	2	9	12	30	114.010	
3	2	4	12	64	116.030	
0	1	14	6	52	116.553	
4	1	0	6	47	117.777	
4	1	3	12	26	121.956	
1	3	10	12	66	127.605	
3	0	12	6	41	129.802	
2	0	14	6	58	131.031	
4	1	6	12	56	135.971	
1	1	15	12	29	142.225	
4	0	10	6	69	145.049	
0	5	4	6	56	149.060	
1	2	14	12	52	149.986	
1	0	16	6	32	150.300	
3	3	0	6	79	152.239	
0	4	20	6	42		80.363
5	1	16	12	28		80.798
7	1	0	6	26		80.998
7	0	10	6	50		82.767
3	3	18	12	36		83.234
1	7	6	12	29		84.097
1	1	24	12	41		84.397
5	4	4	12	32		85.789
6	3	0	6	42		86.120
0	8	4	6	32		88.336
4	2	20	12	26		90.584
2	2	24	12	37		92.038
2	6	14	12	34		93.525
8	1	4	12	32		95.978
3	4	20	12	38		98.243
6	1	20	12	30		103.425
4	6	10	12	36		105.883
7	3	10	12	41		108.546
0	7	20	6	39		108.721
0	0	30	2	44		109.913
5	2	24	12	29		115.794
3	0	30	6	40		118.280
1	9	10	12	26		119.713

## Footnotes

1 Certain commercial equipment, instruments, or materials are identified in this paper to foster understanding. Such identification does not imply recommendation or endorsement by the National Institute of Standards and Technology, nor does it imply that the materials or equipment identified are necessarily the best available for the purpose.
